# Bacterial Nanocellulose Loaded with Bromelain: Assessment of Antimicrobial, Antioxidant and Physical-Chemical Properties

**DOI:** 10.1038/s41598-017-18271-4

**Published:** 2017-12-21

**Authors:** Janaína Artem Ataide, Nathália Mendes de Carvalho, Márcia de Araújo Rebelo, Marco Vinícius Chaud, Denise Grotto, Marli Gerenutti, Mahendra Rai, Priscila Gava Mazzola, Angela Faustino Jozala

**Affiliations:** 1 Graduate Program in Medical Sciences, Faculty of Medical Sciences, University of Campinas, Campinas, SP, Brazil; 2 Department of Technology & Environmental Process, University of Sorocaba, Sorocaba, SP Brazil; 3 LaBNUS – Biomaterials and Nanotechnology Laboratory, University of Sorocaba, Sorocaba, SP, Brazil; 4 Nanobiotechnology Lab., Department of Biotechnology, SGB Amravati University, Amravati, Maharashtra India; 5 Faculty of Pharmaceutical Sciences, University of Campinas, Campinas, SP, Brazil

## Abstract

Bacterial nanocellulose (BNC) has desirable properties for wound healing such as high purity, good shape retention, and high water binding capacity. Bromelain is a protease found in pineapple tissues and has been applied in several fields, it has anti-inflammatory and anticancer properties, promotes cell apoptosis, amongst others. In this work, a BNC based device for the controlled release of bromelain was developed. BNC were submersed in sterilized bromelain solution and incubated at 25 °C under 100 rpm for 24 h. Physical-chemical properties, protein concentration, antioxidant and antimicrobial activities were measured. Results demonstrate that BNC could improve bromelain antimicrobial activity 9 times. Those findings allow concluding that bromelain is a promising molecule to be incorporated into BNC’s. The BNC’s characteristics seem to represent a new promising delivery system of the loaded biomolecule, and protected from external actions.

## Introduction

Current advances in biopolymer research demonstrates their potential for a wide range of applications in pharmaceutical and medical fields, including the development of drug delivery systems, wound healing, scaffolds for tissue engineering, novel vascular grafts, and dressing for skin burns and wound healing^1–4^. The most abundant biopolymer is cellulose, which is produced by a variety of organisms and constitutes the basic structure of cells walls of almost all plants, many *fungi* and some *algae* ^5–7^.

Bacterial nanocellulose (BNC) is an extracellular polysaccharide secreted mainly by *Gluconacetobacter xylinus*, among other bacteria of genera *Gluconacetobacter*, *Agrobacterium Rhizobium, Pseudomonas, Sarcina*, and *Acetobacter* ^8–10^. Unlike vegetable cellulose, BNC is produced in pure form, free of other polymers. In addition, it can be molded into three-dimensional structures, able to retain high water amounts, mechanically resistant and biocompatible^11^. BNC has a nanofibrilar structure, making it an ideal matrix for use as medical devices, either as an aid in healing skin lesions^3^ or in tissue engineering, assisting cell regeneration^12,13^.

The scope of applications of BNC can be further expanded through its association with bioactive molecules. For instance, it has been shown that the incorporation of antimicrobial agents into BNC membranes yielded an active packaging system^14^. Nisin-containing BNC films were effective in controlling *L. monocytogenes* ^15^, while BNC containing sorbic acid was used as a food packaging material with an ability to controlled release the antimicrobial agent^16^.

Proteolytic enzymes could be used as therapeutics adjuvants, due to their debridement and antimicrobial activities, facilitating tissue renewal process and accelerating ulcerated skin healing. Bromelain is a set of proteolytic enzymes found in pineapple (*Ananas comosus*) tissues such as stem, fruit and leaves, and has widespread applications, such as in the cosmetic and food industries, as well as in medicine^17–19^. In healthcare, bromelain has been used for the treatment of rheumatoid arthritis, thrombophlebitis, hematomas, oral inflammation, diabetic ulcers, rectal and perirectal inflammation, wounds, inhibition of platelet aggregation, angina pectoris, bronchitis, sinusitis, surgical traumas, and pyelonephritis, and for enhancing the absorption of drugs, particularly antibiotics^18,20–22^.

Dutta and Bhattacharyya^23^ reported that bromelain from pineapple crown leaves present nonspecific proteolytic, gelatinolytic, collagenase, fibrinolytic, acid and alkaline phosphatase, nuclease, peroxidase, with considerable anti-bacterial and anti-fungal activities. In a further study using specific cysteine proteases inhibitors, authors found that papain and bromelain antibacterial activity is not related to its proteolytic activity, and may be related to amidase and esterase activities^24^.

Collectively, these bromelain properties support its application in the treatment of microbial infections and also as a wound healing agent^25^. Thus, the incorporation of bromelain in BCN membranes could be of potential interest in different fields, mainly in pharmaceutical and medical applications, such as the development of a delivery system for healing application.

This article describes bromelain loading into BNC membranes and its release. Also presents physical-chemical properties, protein concentration, antioxidant and antimicrobial activities of developed system (Supplementary Fig. S1).

## Results and Discussion

### Bromelain loading and release from bacterial cellulose membrane

The many advantages of BNC including its biocompatibility, conformability, elasticity, transparency, ability to maintain a moist environment in the wound, and ability to absorb exudates during the inflammatory phase, provide great potential for applications in wound healing systems^26,27^. The novelty in this study is the incorporation of bromelain in BCN membranes, to improve the antimicrobial characteristics and create a potential interest in different fields, mainly in pharmaceutical and medical applications.

**Figure 1:**
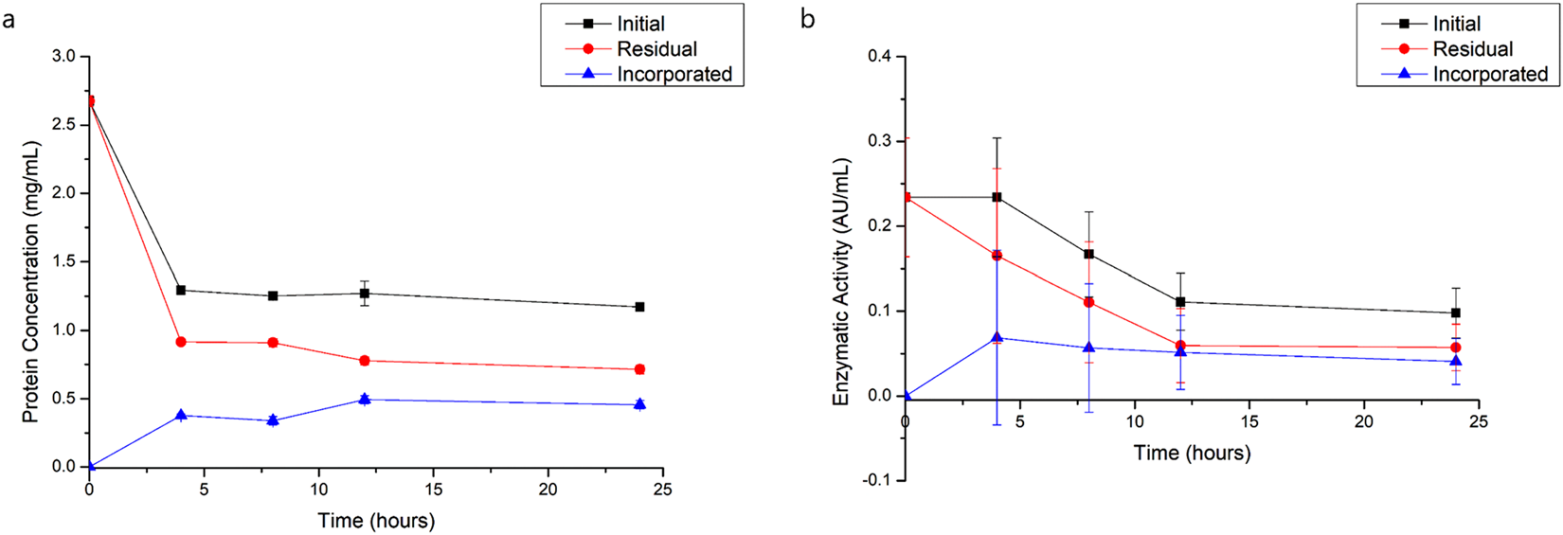
Protein concentration (**a**) and enzymatic activity (**b**) of initial and residual bromelain solutions, and bacterial nanocellulose membrane, where error bars correspond to standard deviation.

Bromelain was incorporated in BNC membranes by immerging membranes in a bromelain solution. Along with incubation time with the BNC membrane, bromelain enzymatic activity in the supernatant decayed (Fig. 1), indicating enzyme migration into the membrane. Bromelain solution was subjected to the same loading conditions, but without contact with the bacterial cellulose, and there was an enzymatic activity and protein decrease, which was considered to calculated bromelain loading into BNC membranes. These results show that bromelain migrated to the interior of BNC.

Bernkop-Schnürch, *et al*.^28^ used bromelain as a model in the study of incorporation and release of microparticles of poly(acrylic acid) over a period of 60 minutes at 37 °C. Additionally, the bromelain release rate could be controlled through the selection of the hydrophobicity of the polymers.

**Figure 2:**
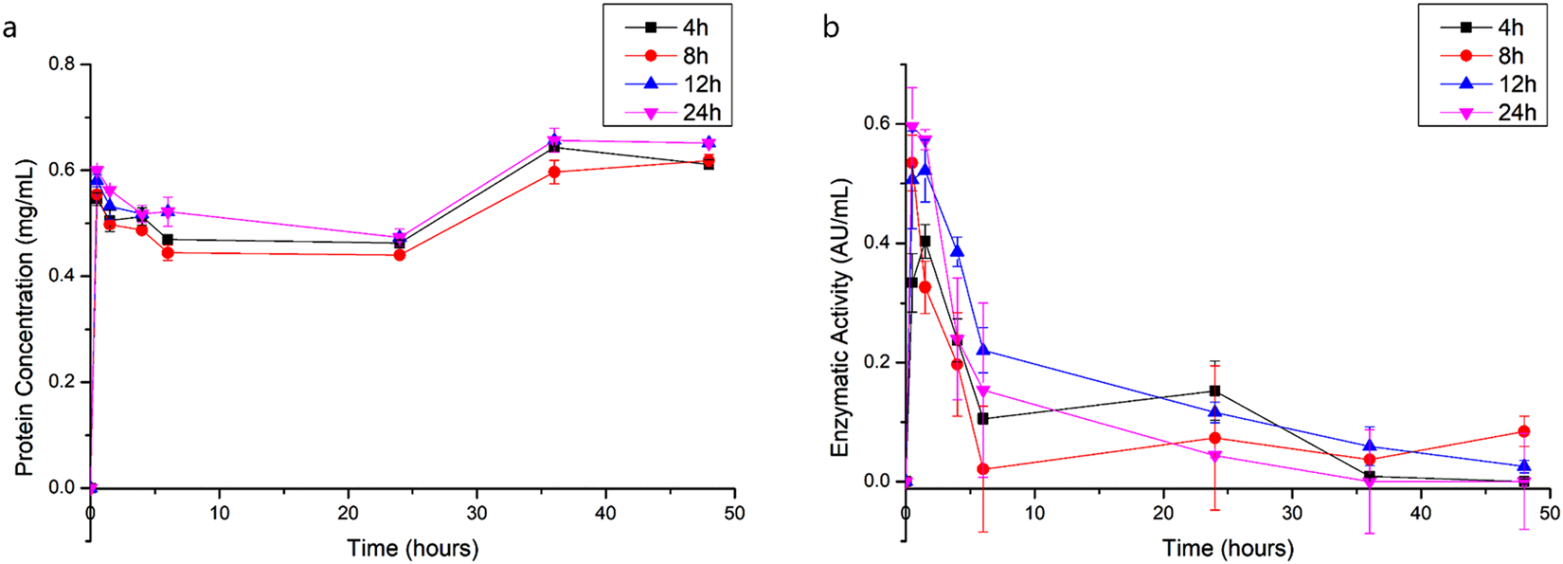
Protein concentration (**a**) and enzymatic activity (**b**) of released bromelain from bacterial cellulose membranes after different incorporation times at 25 °C, where error bars correspond to standard deviation.

After the first 30 minutes, released bromelain amount was higher than the incorporated amount, for protein concentration and enzymatic activity (Fig. 2, Supplementary Fig. S2). The same results were observed by^19^, when incorporating bromelain into alginate and Arabic gum hydrogel. According with suppliers, commercial steam bromelain purity is 35%, and it contains other components with high molecular weight^29^, which may be not incorporated by BNC. Thus, it is possible to infer that BNC, as well as the alginate and Arabic gum based hydrogel^19^, selectively absorbed low molecular weight bromelain components, which present higher enzymatic activity.

### BNC structural analysis by Scanning Electron Microscopy

**Figure 3:**
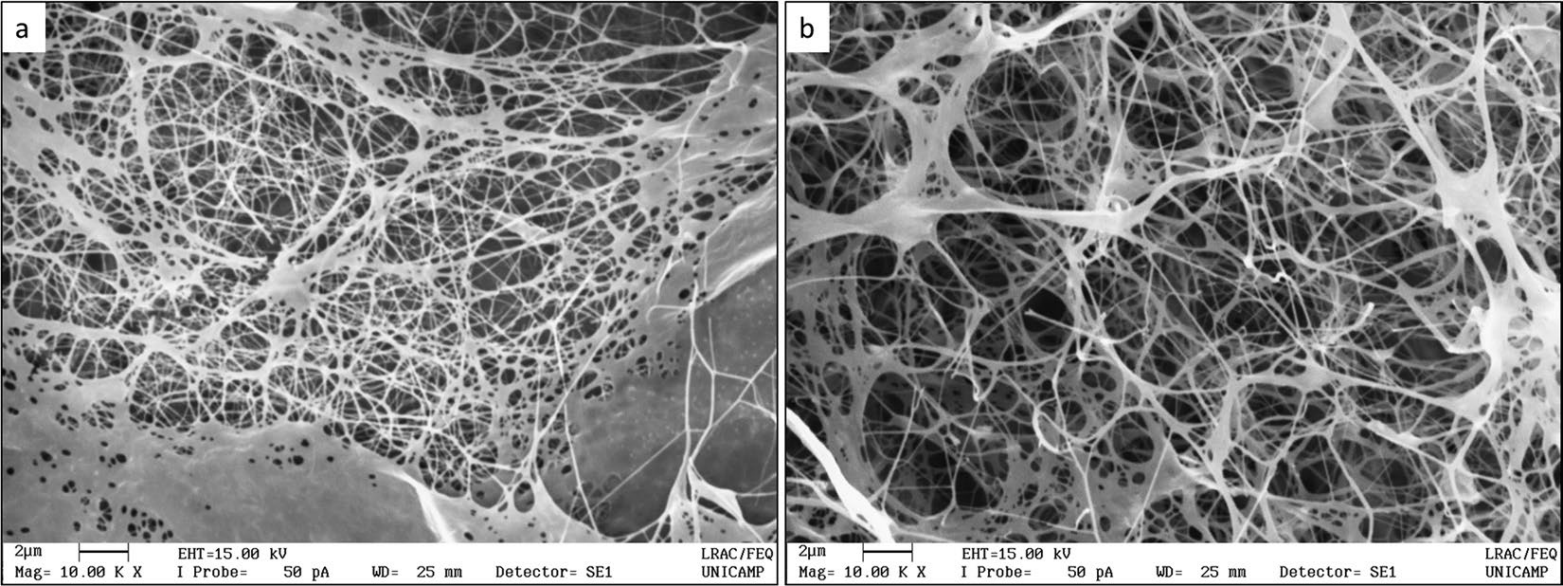
Scanning electron microscopy of bacterial nanocellulose membranes (**a**) and bromelain-loaded membranes (**b**).

SEM analyses, done at 10 kx magnifications, indicated that the BC membranes presented an entangled structure, with void spaces randomly distributed throughout the membrane matrix (Fig. 3). The highly entangled thin fiber network accounts for a large surface area and the porous structure of the BNC membranes facilitates the entrapment and immobilization of proteins^30^. However, no relevant changes in BNC network was observed when bromelain was loaded, which was similar to previous results reported by Müller, *et al*.^31^ and Moritz, *et al*.^32^.

### FTIR spectroscopy

**Figure 4:**
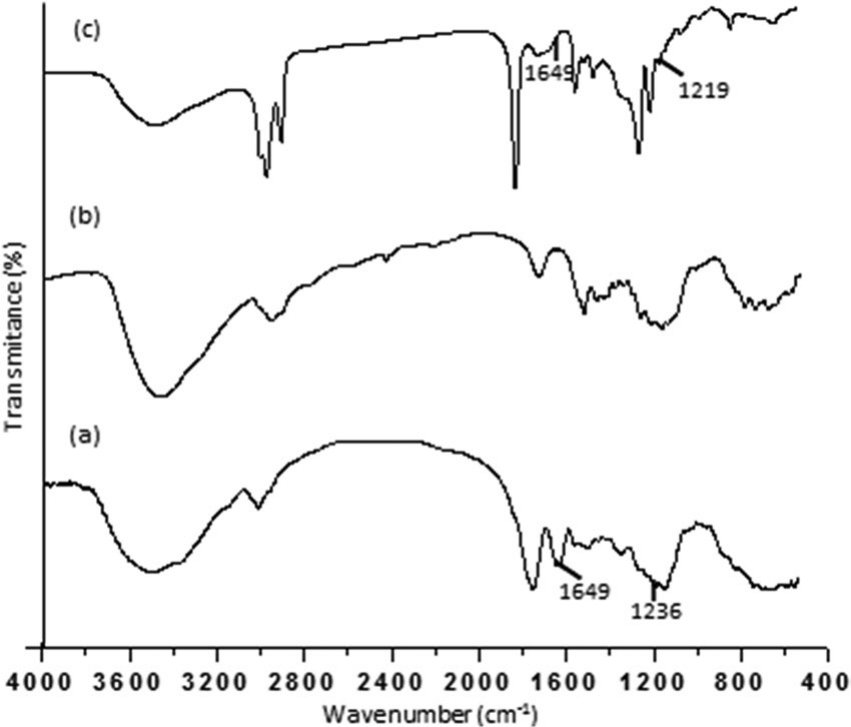
FTIR spectra of pure bromelain (**a**), bacterial cellulose membrane (**b**) and bacterial cellulose membrane with bromelain (**c**).

Chemical cleavages characteristic of pure bromelain and bacterial cellulose membranes without and with bromelain were determined by Fourier Transform Infrared (FTIR) spectroscopy shown in Fig. 4. The bromelain-containing bacterial cellulose membrane showed a vibration displacement at 1649 cm^−1^, relative to the C grouping=O. The bromelain bacterial cellulose membrane showed a vibration displacement at 1649 cm^−1^ related to the C=O band. The C=O stretching group corresponds to the amide group from asparagine, glutamine amino acids or residual proteins of BCN membrane. The stretching in 1612 cm^−1^ can be attribute at N-H from amide I group of bacterial cellulose protein^33^. The aliphatic amine (1236 cm^−1^) is observed at 1219 cm^−1^ on the bromelain-containing bacterial cellulose membrane^33^. The stretching in the range of 3200–3800 nm^−1^ for bromelain (a), BCN membrane (b) and BCN with bromelain (c) is corresponding to -OH group. The decrease in OH stretching is related to the formation of hydrogen bonding between immobilisation of bromelain that occurs through the establishment of hydrogen bonding between -OH groups on the surface of -NH_2_ of BNC membrane^19^. Bromelain interaction with BCN membranes may also be attributed to hydrophobic interactions or Van der Waals forces, once bromelain contains hydrophobic amino acids exposed on its surface^34,35^.

### Evaluation of bromelain antimicrobial activity

For minimal inhibitory concentration (MIC), the following solutions were testes: (i) initial bromelain solution; (ii) residual bromelain solution; and (iii) bromelain solution after being released from BCNs. Table 1 present protein concentration, enzymatic activity and specific activity of those solutions.

**Table 1:** Total protein concentration, enzymatic and specific activity of bromelain solution in initial condition and after BCN membranes loaded and release.

	Protein Concentration (mg/mL)	Enzymatic Activity (U/mL)	Specific Activity (U/mg)
Initial Solution	25.57	8.7	0.34
Residual Solution*	17.63	6.78	0.38
Release Solution*	1.43	1.42	0.99

*Collected after 24 hours.

It is possible to note that residual bromelain solution presented a decrease in all parameters when compared with initial solution, indicating that BCN membrane was capable to absorb 7.94 mg/mL of proteins, representing 31% of total protein concentration in the initial bromelain solution. It is also possible to observe, an increase in bromelain specific activity in the release solution (3-times higher than initial solution), which may be related with BCN membrane selectively absorbing bromelain low molecular weight components with higher enzymatic activity.

**Figure 5:**
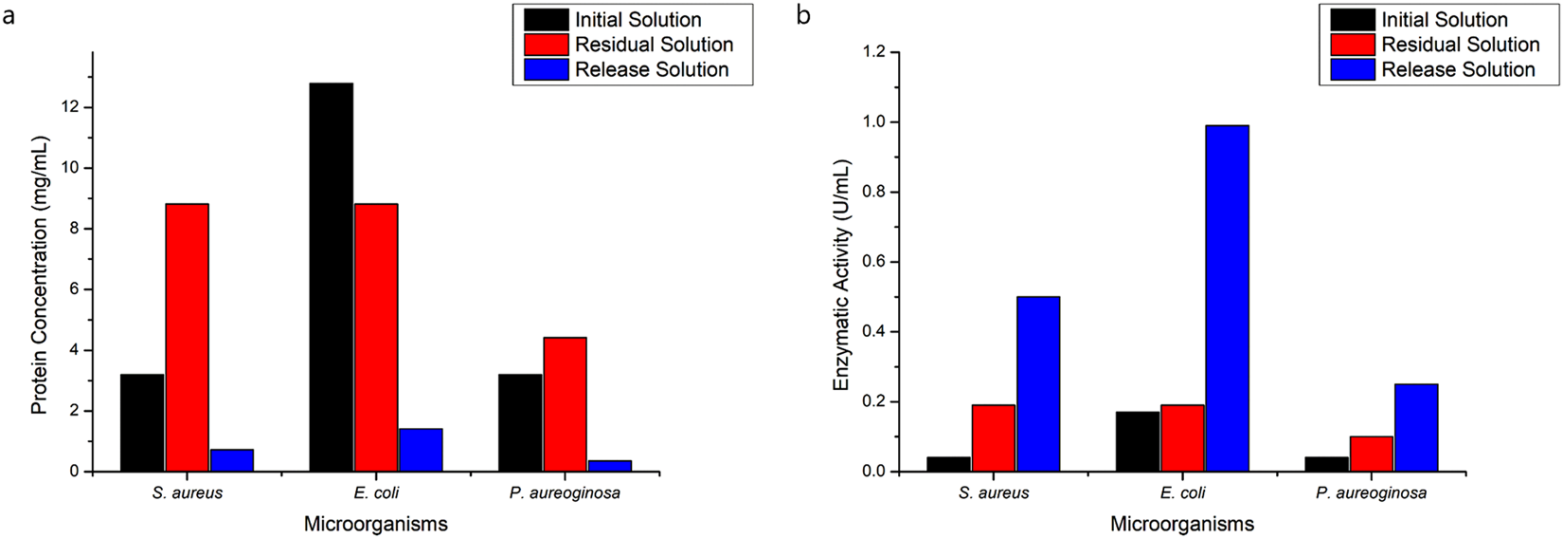
Protein concentration (**a**) and enzymatic activity (**b**) of initial and residual bromelain solution, and release solution found as MIC.

Those three solutions, were used for the minimal inhibitory concentration assay (MIC), Fig. 5 presents protein concentration (Fig. 5a) and enzymatic activity (Fig. 5b) of tested solutions when MIC was achieved.

Results showed in Fig. 5b indicate the bromelain antimicrobial activity has a relation with its enzymatic activity. It is possible observe this effect for all microorganism, to *E. coli*, bromelain release solution MIC was 0.99 U/mL, 6 times higher than initial solution MIC. The same behavior was observed for *P. aureoginosa*, where MIC was achieved at 0.25 U/mL in the release solution, 6 times higher than MIC of initial solution 0.04 U/mL, and for *S. aureus* bromelain release solution MIC was 0.5 U/mL, 12 times higher than initial solution MIC.

According to Ali, *et al*.^36^, crude bromelain is more effective in inhibiting Gram-positive bacteria growth than Gram-negative. Our results, however, pointed in a different direction, since bromelain inhibit all tested microorganisms, especially for release solution. Dutta and Bhattacharyya^23^ reported bromelain antimicrobial activity against *S. aureus*. A bromelain solution with 1.95 mg/mL concentration inhibited *S. aureus* growth by 50%, however bromelain enzymatic activity was not reported. The same study showed the bromelain concentration of 1.86 and 2.11 mg/mL were responsible to inhibit *E. coli XL1 blue* and *E coli DH5α* Pet16b (Amp)^r^ growth, respectively. Our results (Fig. 5a) showed a higher protein concentration for bromelain initial solution (3.2 mg/mL) and lower concentration for release bromelain solution (0.72 mg/mL) when compared to previous study, which also could be explained by the high specific activity of bromelain after incorporated in BNC membranes.

Crude bromelain extract (1.8 mg/mL) and standard bromelain (2 mg/mL) created a 22 mm zone of inhibition of *E. coli* growth at 37 °C in neutral pH. However, at alkaline pH, crude bromelain extract and standard bromelain created a zone of growth inhibition of 8.33 and 9.67 mm, respectively^36^. In our study, initial bromelain solution and release solution MIC was 12.79 mg/mL and 1.41 mg/mL concentration, and enzymatic activity was 0.17 AU/mL and 0.99 AU/mL, respectively. In this case, it was also observed a lower protein concentration with higher enzymatic activity for release solution.

*Aggregatibacter actinomycetemcomitans*, *Porphyromonas gingivalis*, and *Streptococcus mutans* have been predominantly associated with periodontal diseases. Bromelain was investigated as a potential antimicrobial agent to be used as a monotherapy and as an adjunct with mechanical debridement. Bromelain showed antibacterial efficacy against all the isolated strains of both aerobic and anaerobic microorganisms. *S. mutans* showed sensitivity at the lowest concentration (2 mg/mL), followed by *P. gingivalis* (4.15 mg/mL). While *A. actinomycetemcomitans* demonstrated the higher resistance and a MIC of 16.6 mg/mL^37^.

**Figure 6:**
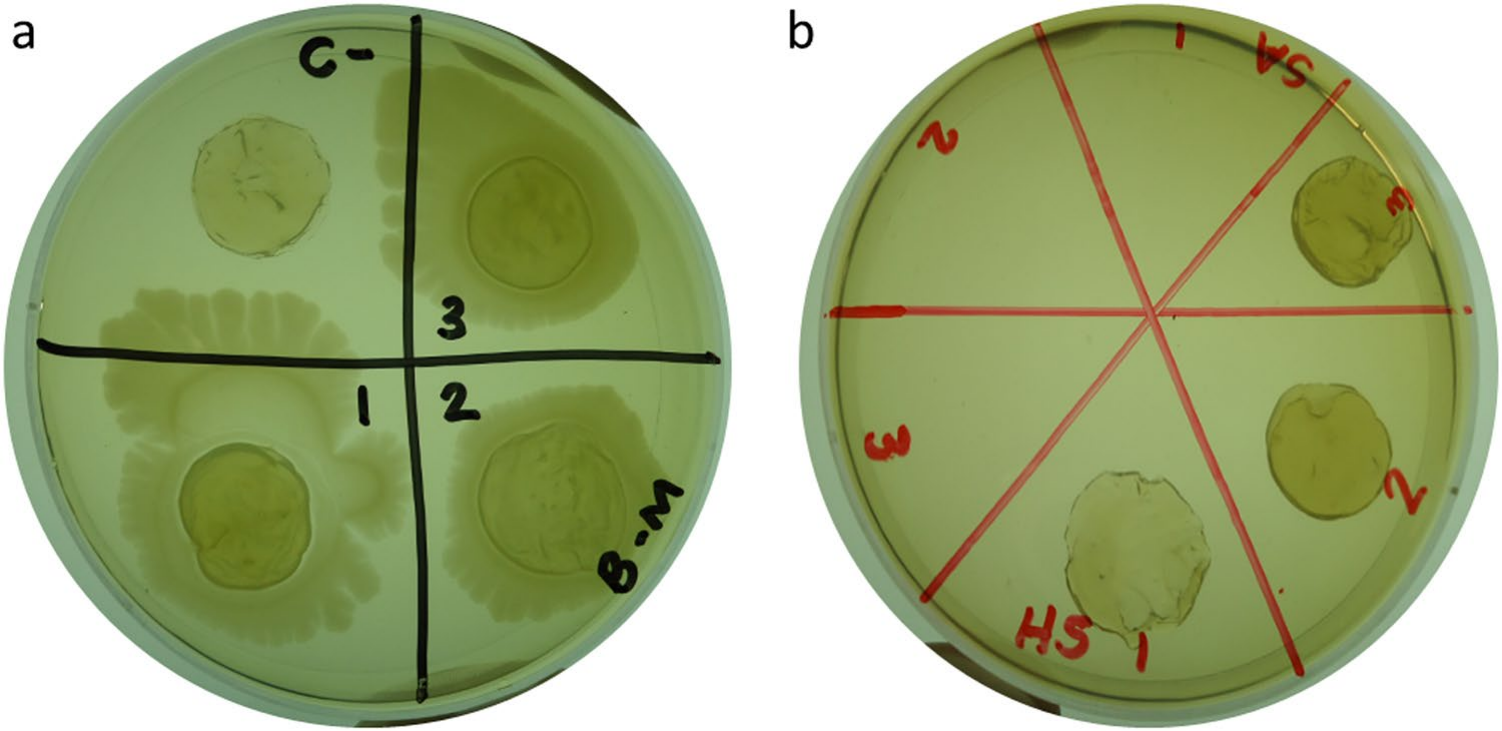
Antimicrobial activity evaluated by modified agar diffusion method of bacterial nanocellulose membranes (**a**) and bromelain-loaded membranes (**b**) for *S. aureus* (1), *E. coli* (2) and *P. aeruginosa* (3).

For agar diffusion modified assay, bromelain-loaded BNC membranes showed antimicrobial activity for all tested microorganisms (Fig. 6b), differently from BNC membranes without bromelain (Fig. 6a).

### Bromelain antioxidant activity

Proteases enzymes, such as bromelain and papain, present antioxidant activity as free radical scavenging and lipid peroxidation inhibition^38^. Bromelain antioxidant activity was measured against 1,1-diphenyl-2-picrylhydrazyl (DPPH) radical, which provides an easy and rapid way to evaluate antiradical activities^39^.

**Figure 7:**
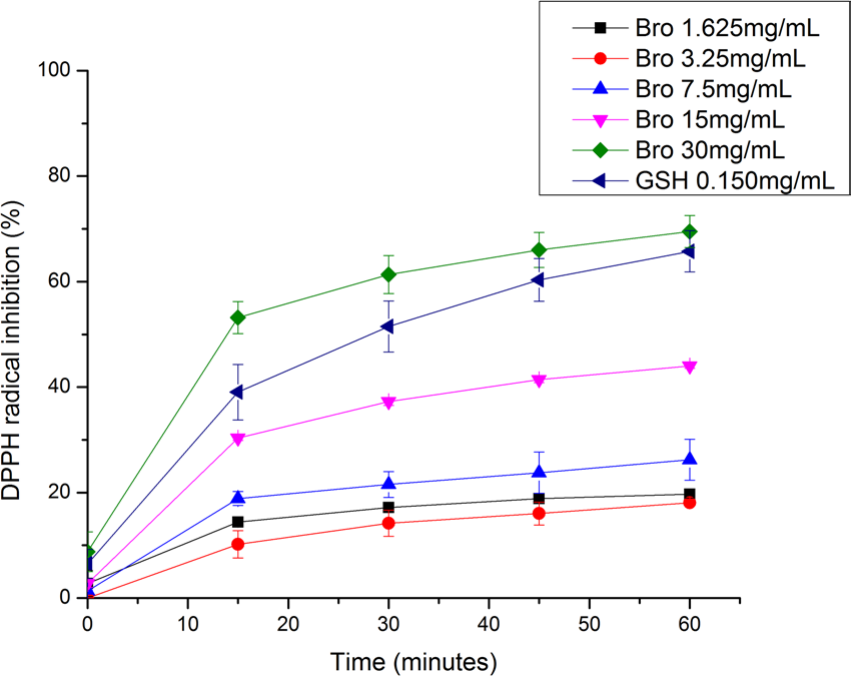
Percent of 1,1-diphenyl-2-picrylhydrazyl (DPPH) radical inhibition by the bromelain (Bro, in different concentrations) by the reduced glutathione (GSH), where error bars correspond to standard deviation.

Figure 7 shows the antioxidant activity of different concentrations of bromelain initial solution over 60 minutes. According with our results, bromelain antioxidant activity was dose-dependent, and so the highest bromelain concentration (30 mg/mL) presented the highest antioxidant activity, in the lowest reaction time. At the lowest concentration (1.625 mg/ml) antioxidant activity was not significant (p > 0.05).

Both bromelain at 30 and 15 mg/mL were statistically similar to glutathione (GSH), antioxidant standard (0.150 mg/mL), initially and after 15 minutes. However, over time, bromelain at 15 mg/mL showed a statistically lower antioxidant activity than GSH (p < 0.05) and bromelain at 30 mg/mL (p < 0.05). It is important to observe that antioxidant capacity of bromelain increased over time even in the lowest concentrations, a behavior also seen in GSH. This feature is relevant mainly when considering a formulation for topical application for example, in which the antioxidant activity remains and even increases after its application.

Several studies have been reported about bromelain and its medical use, such as inhibition of platelet aggregation and fibrinolysis^40^, effects on cardiovascular diseases^41^, modulation of inflammatory system^42^ and skin debridement properties^43^. Among them, the latter two are very convenient when associated to the antioxidant and antimicrobial capacity found in this study, once these four properties are essential in skin lesions, as a wound healing.

### Mucoadhesive property

Mucoadhesive property was evaluated by a tensile test, and is a property related to the ability of a material (synthetic or biological) to adhere to a biological tissue during a certain period of time^44^. In this way, mucoadhesion analysis evaluates the interaction between the membrane and the surface of the mucosa at a temperature of 37 °C^45,46^.

Analysis of the results shows that maximum strength values significantly (p < 0.05) reduced from 0.493 ± 3.6 N to 0.112 ± 0.1 N, respectively for bacterial cellulose membrane without and with bromelain. According to Yadav, *et al*.^44^, mucoadhesion may occur by increasing the viscosity of the system or by molecular interactions, such as ionic interactions or formation of hydrogen (OH) bonds. It is worth mentioning that the immobilization of bromelain in the bacterial cellulose membrane occurs by the formation of hydrogen bonds between OH of bacterial cellulose membrane and bromelain NH_2_^47^. In this way, one can suggest that the decrease of the mucoadhesive property occurred by the decrease of free OH groups in the membrane of bacterial cellulose containing bromelain, results evidenced in the FTIR analysis.

## Conclusion

BCN membranes were able to selectively absorb and release bromelain. Bromelain itself present antimicrobial and antioxidant activities, however, after incorporated into BNC membranes, its antimicrobial activity increased. BNC membranes presented mucoadhesive properties, which decreased after bromelain loading. This can be explained by the presence of H-bonds between bromelain and BNC membrane, leading to a decrease in molecular interactions between BNC and mucin. The broad bromelain solution and BNC membrane antimicrobial spectrum and their tissue adhesion make them an alternative method for wound contamination control, since the membrane may act as platform that delivers antimicrobial gradually avoiding biofilm formation. Thus, bromelain-containing BNC membrane proved to be a promising drug delivery system to minimize healing period, reducing the risk of infection and promoting patient comfort and pain relief.

## Materials and Methods

### Materials

Bromelain, Folin-Ciocateau and bicinchoninic acid assay kit were purchased from Sigma-Aldrich (Brazil). Other reagents were of analytical or food grade. Assays were performed in triplicate.

### Production and purification of bacterial cellulose membranes

Bacterial nanocellulose membranes (BCN membrane) were produced through *Gluconacetobacter xylinus* (ATCC 53582) cultivation in Hestrin & Schramm medium (20 g/L glucose, 5 g/L bacteriological peptone, 5 g/L yeast extract, 2.7 g/L anhydrous sodium phosphate, 1.5 g/L monohydrate citric acid). Microorganism growth in 24-well cell culture plates, containing 1 mL of a 10^6^ CFU/mL inoculum per well.

**Figure 8:**
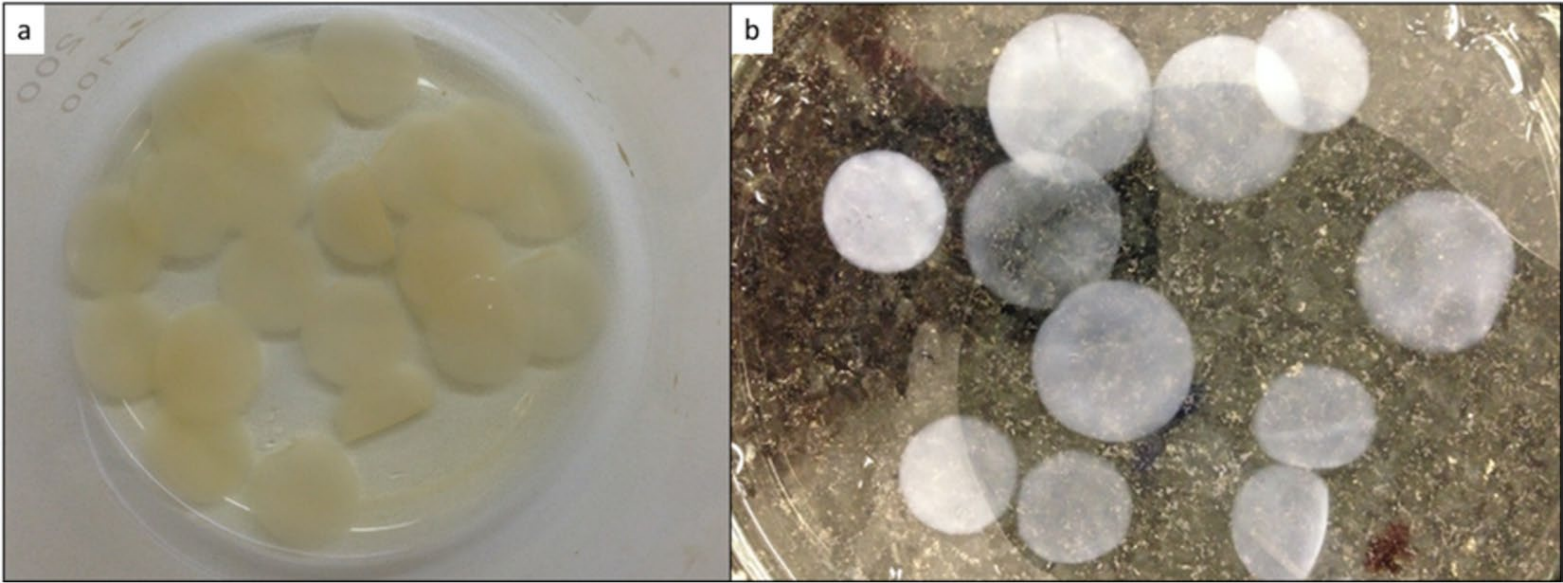
Bacterial nanocellulose membrane (BCN membrane): appearance after production (**a**) and after NaOH treatment and sterilization (**b**).

Plates were kept for 4 days in static culture at 30 °C, yielding 2 mm thick membranes. After growth, membranes were washed in 2% sodium dodecyl sulfate (SDS) solution overnight, rinsed with distilled water until SDS removal, and immersed in 1 M NaOH solution with stirring at 60 °C for 1 and a half hours. After this period, membranes were washed again until reaching neutral pH. Then they were packaged and autoclaved at 121 °C for 15 minutes in Milli Q water, and stored at 4 °C (Fig. 8).

### Enzymatic activity and protein quantification

Enzymatic activity was determined by adapting the non-specific protease activity assay from Sigma^®^^48–50^. Briefly, the method consists in casein cleavage by bromelain for 10 minutes at 37 °C and pH 7.5 and the reaction was stopped by adding trichloroacetic acid (TCA). The resulting solution was centrifuged at 4,000 g for 20 minutes, and a supernatant aliquot placed to react with Folin-Ciocalteau, in the presence of sodium carbonate. The reaction of Folin primarily with tyrosine produces free chromophore with a blue color, which is measured by absorbance analysis at 660 nm. The standard curve was drawn with L-tyrosine. Protein concentration was determined with bicinchoninic acid assay^51^.

### Bromelain loading in bacterial cellulose membranes

In 24-well plates, BNC membranes were disposed and 1 mL of 30 mg/mL bromelain solution in citrate-phosphate buffer (pH 5.0) was poured into the plate. In the fourth well only bromelain solution was added. Plates were kept in 100 rpm orbital shaker at 25 °C for various periods ranging for 4, 8, 12 and 24 hours. After each period, supernatants were collected and bromelain activity and protein quantification were evaluated in the isolated residual solutions.

### Bromelain release from bacterial cellulose membranes

This methodology was used to assess bromelain release from BNC membranes. In 24 well plates, membranes loaded with bromelain (after 24 hours loading time) were disposed in triplicate, with a negative control (membrane without bromelain) in the fourth well. In each well 1 mL of citrate-phosphate buffer (pH 5.0) was added. Plates were placed in 100 rpm orbital shaker at 25 °C. At different periods (0, 0.5, 2, 4, 6, 24, 36 and 48 hours), membranes and supernatant were collected. Bromelain activity and protein quantification were assayed in the isolated released solution.

### Scanning Electron Microscopy

BNC membranes structure was observed using a Leo 440i scanning electron microscopy with 6070 X-ray dispersive energy detector (LEO Electron Microscopy, England). SEM images were obtained using an accelerating voltage of 15 kV. Before analysis, samples were lyophilized for 24 hours and coated with gold (92 A°) using SC7620 Sputter Coater POLARON (VG Microtech, England).

### Fourier Transform Infrared Spectroscopy (FTIR)

The free bromelain and bacterial cellulose membrane with and without bromelain were analysed using Fourier Transform Infrared Spectroscopy (FTIR) (Shimadzu, FTIR IRAffinity-1S, Kyoto, Japan) using transmittance modes. Membranes were kept in an oven at 30 °C, after drying they were ground. Approximately 2 mg of the sample was mixed with 300 mg of KBr to form the pellet. Spectra were obtained in the 4000 to 400 cm^−1^ wavelength range after 64 scans, with a resolution of 4 cm^−1^. The spectra were normalized and the vibration bands were associated with the main chemical groups.

### Evaluation of bromelain antimicrobial activity

In this assay, the microorganisms used were *Escherichia coli* (ATCC 25922) and *Pseudomonas aeuroginosa* (ATCC 9721), representing the class of gram-negative; and *Staphylococcus aureus* (ATCC 10390), representing the class of gram-positive.

#### Minimum inhibitory concentration

Minimum inhibitory concentration (MIC) classical method of successive dilution was adapted to 96-well plates, in triplicate^52,53^, using (i) initial bromelain solution; (ii) residual bromelain solution; and (iii) bromelain solution after being released from BCNs.

#### Agar diffusion assay

The agar diffusion assay was modified to verify qualitatively the antimicrobial activity in BCN membranes itself and after bromelain loading^54,55^. Membranes were placed in 24-well plates with 1 mL of culture medium and 100 μL of the microorganism suspension (10^6^ CFU.mL^−1^), and plates incubated for 24 hours at 37 °C. After this time, the resulting membrane and solution were inoculated intro Petri dishes containing agar of the culture medium.

### Bromelain antioxidant activity

Antioxidant activity was determined based on absorbance decrease at 515 nm after the reaction between radical 1,1-diphenyl-2-picrylhydrazyl (DPPH) and initial bromelain solutions, according to Brand-Williams, *et al*.^39^.

### Mucoadhesive strength proprieties

Mucoadhesive strength of the bacterial cellulose membrane with and without bromelain was evaluated by the force required to remove the membrane from a mucin disc using the TAXTPlus texture analyzer (Stable Micro Systems, UK). Samples were fixed in a water bath with a temperature set at 37 °C. The mucin discs were prepared by compression (Lemaq, rotary compressor machine, Mini Express LM-D8, Diadema, BR). The surface of the mucin disc was hydrated and the disk attached to the lower end of the analytical probe. Mucin disc was compressed apical → basal on the surface of the samples, with a compression force of 0.1 N. The contact time of the disc with the surface of the samples was 200 s. Removal of the analytical probe was at the rate of 0.5 mm.s^−1^. The force required to separate the surfaces was determined by time *versus* force ratio (n = 3), and analysis parameters were described on Table 2.

**Table 2:** Parameters used to evaluate the mucoadhesive property of bacterial cellulose membrane with and without bromelain.

	Parameters
Aparate	Film Support Rigg Part Code. HDP/FSR Batch n 13085
Test Mode	Compression
Pre-Test Speed	0,5 mm/s
Test Speed	0,5 mm/s
Post-Test Speed	10 mm/s
Target Mode:	Distance
Distance	—
Trigger Type	Auto
Trigger Force	0,049 N

### Statistical analysis

Data is presented as mean ± standard deviation. Differences among bromelain concentrations were evaluated by one-way ANOVA, followed by Tukey multiple range tests. P values < 0.05 were considered significant. Data were analyzed using Statistica®8.0 (Statsoft software, Tulsa, OK, USA).

## Electronic supplementary material

Supplementary Information
