# Supplementary material for: Bacterial Nanocellulose Loaded with Bromelain: Assessment of Antimicrobial, Antioxidant and Physical-Chemical Properties
Source: Sci Rep. 2017 Dec 21;7:18031. doi: 10.1038/s41598-017-18271-4 (PMC5740125; doi:10.1038/s41598-017-18271-4)
Supplement: Supplementary file 1 — Supplementary Information [file 41598_2017_18271_MOESM1_ESM.pdf]

Bacterial Nanocellulose Loaded with Bromelain: Assessment of Antimicrobial, Antioxidant and Physical-Chemical Properties

Janaína Artem Ataide[1], Nathália Mendes de Carvalho[2], Marcia de Araujo Rebelo[3], Marco Vinícius Chaud[3], Denise Grotto[2], Marli Gerenutti[2], Mahendra Rai[4], Priscila Gava Mazzola[5], Angela Faustino Jozala[2]

[1]Graduate Program in Medical Sciences, Faculty of Medical Sciences, State University of Campinas, Campinas, Brazil
[2]Department of Technology & Environmental Process, University of Sorocaba UNISO, Sorocaba, SP – Brazil
[3]LaBNUS – Biomaterials and Nanotechnology Laboratory, i(bs)2 – intelligent biosensing and biomolecule stabilization research group, University of Sorocaba, Sorocaba/SP, Brazil
[4]Nanobiotechnology Lab., Department of Biotechnology, SGB Amravati University, Amravati, Maharashtra, India
[5]Faculty of Pharmaceutical Sciences, State University of Campinas, Campinas, Brazil

CORRESPONDING AUTHOR:

Janaína Artem Ataide – Graduate Program in Medical Sciences, Faculty of Medical Sciences, University of Campinas (Unicamp), Campinas/SP, Brazil, zip code 13083-887

Phone Numbers: +55 19 99777 2620 / +55 19 3521 8884

E-mail Address: janaina.a.ataide@gmail.com

Postal Address: Faculty of Medical Sciences, University of Campinas (Unicamp) - Tessália Vieira de Camargo Street, 126, Cidade Universitária "Zeferino Vaz" - Campinas - SP - Brasil – zip code: 13083-887

SUPPLEMENTARY INFORMATION

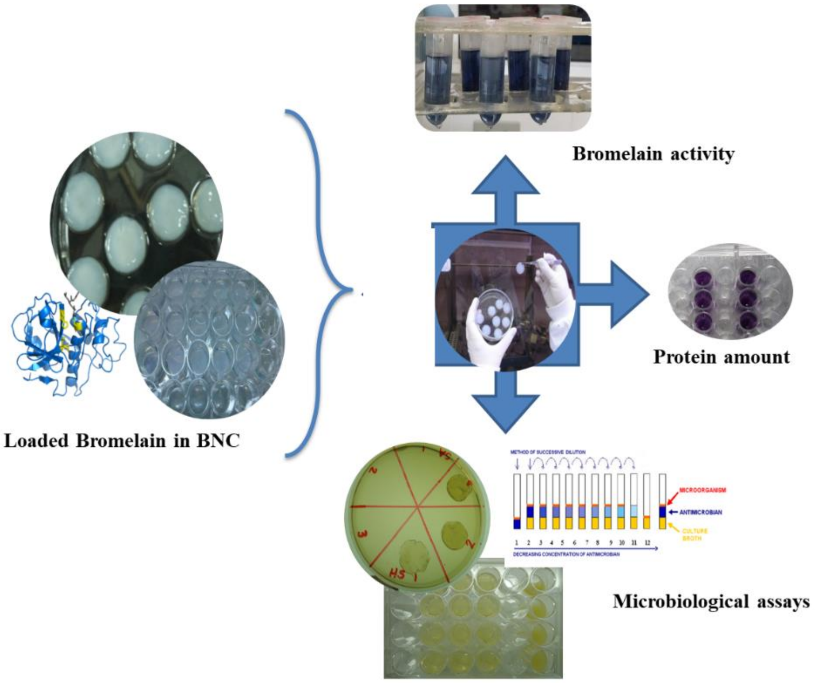

Supplementary Figure S1. Graphical scheme for general understanding of the manuscript.

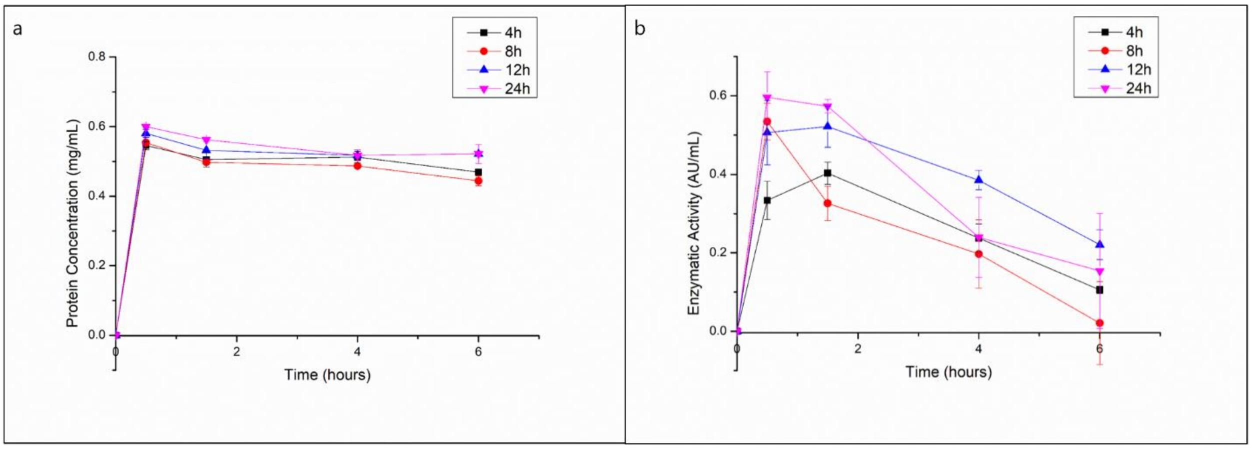

Supplementary Figure S2. Protein concentration (a) and enzymatic activity (b) of first 6 hours of bromelain release from bacterial cellulose membranes after different incorporation times at 25°C, where error bars correspond to standard deviation. Figure presenting time from 0 to 6 hours, enhancing the release in this time, once it is not so clear in figure 2 of main manuscript.
